# Developing an Interactive Human Papillomavirus Vaccination Education Program for Oropharyngeal Cancer Patients and Healthcare Providers: Lessons Learned from the Target Users’ Feedback

**DOI:** 10.3390/vaccines14080689

**Published:** 2026-08-11

**Authors:** Cameron Andrew Jernigan, Hope Reeves, Bradley McNeese, Cate Moriasi, Greg Krempl, Joan Walker, Ashlea Braun, Radhika Gogoi, Thanh C. Bui

**Affiliations:** 1 TSET Health Promotion Research Center, Stephenson Cancer Center, University of Oklahoma Health Campus, Oklahoma City, OK 73104, USA; cameron-jernigan@ou.edu (C.A.J.); hope-reeves@ou.edu (H.R.); bradley-mcneese@ou.edu (B.M.); cate-moriasi@ou.edu (C.M.); ashlea-braun@ou.edu (A.B.); 2 Department of Family and Preventive Medicine, College of Medicine, University of Oklahoma Health Campus, Oklahoma City, OK 73104, USA; 3 Department of Otolaryngology, College of Medicine, University of Oklahoma Health Campus, Oklahoma City, OK 73104, USA; greg-krempl@ou.edu; 4 Department of Gynecologic Oncology, College of Medicine, University of Oklahoma Health Campus, Oklahoma City, OK 73104, USA; joan-walker@ou.edu; 5 Karmanos Cancer Institute Division of Oncology, Wayne State University, Detroit, MI 48201, USA; radhikagogoi@wayne.edu

**Keywords:** human papilloma virus, oropharyngeal cancer, vaccination, prevention

## Abstract

**Background**: Human papillomavirus (HPV) is associated with multiple head and neck cancers, many of which are preventable with the 9-valent HPV vaccine. We developed an interactive HPV vaccination education program (IHVEP) to equip patients with HPV-associated cancers to serve as health educators. **Objective**: To pilot test the adapted IHVEP, evaluate its content using the Information-Motivation-Behavioral Skills (IMB) model and the Suitability Assessment of Materials (SAM) instrument, and identify factors relevant to optimizing digital health interventions. **Methods**: A previously developed cervical cancer–focused IHVEP was adapted for patients with oropharyngeal cancer. Participants completed the program and participated in semi-structured interviews. Feedback was analyzed and organized into themes aligned with the IMB and SAM frameworks. **Results**: Participants generally perceived the adapted IHVEP as informative, motivating, and empowering. Key motivators included physician recommendation and a desire to protect others. The program appeared to enhance participants’ perceived ability and self-efficacy to promote HPV vaccination. Modules were described as clear, well-organized, linguistically accessible, and culturally appropriate. Feedback identified areas for improvement related to technology, content, and functionality. **Conclusions**: Findings suggest that effective digital health interventions may benefit from incorporating training in evaluating health information, ensuring intuitive design and navigation, and including content featuring real individuals. These results will inform further refinement of IHVEP and the development of future digital health interventions.

## 1. Introduction

Human Papillomavirus (HPV) is the most prevalent sexually transmitted disease in the United States (US), with 85% of sexually active individuals contracting HPV during their lifetime [1]. While some strains of HPV only cause genital warts, other malignant serotypes cause several types of precancer and cancer. According to the US Centers for Disease Control and Prevention (CDC), high-risk HPV causes 91% of cervical cancer, 70% of oropharyngeal cancer, and approximately 92% of all HPV-associated cancers could be prevented by 9-valent HPV vaccination [2].

The US CDC recommends HPV vaccination for everyone aged 11–26 years [3]. Although the HPV vaccine has proven effective in preventing HPV-associated cancers, vaccine uptake has slowed nationwide, largely due to rising vaccine distrust following the COVID-19 pandemic [4,5]. Although the most predictive factor influencing HPV vaccination acceptance is the recommendation of an authoritative provider, interventions based on this strategy have not effectively increased vaccination rates [6,7]. Parental concerns about vaccine safety have increased in recent years, even as actual adverse event data remained stable [8]. This association suggests traditional educational interventions have been ineffective in combatting vaccine misinformation. Traditional approaches to promoting HPV vaccination by educating eligible individuals or their parents have limited success [9]. Thus, there is a pressing need to develop novel approaches designed to improve public perception of the HPV vaccine.

Engaging patients as health educators within their social networks has been shown to be effective for addressing misinformation and promoting disease prevention across multiple populations and health contexts [10,11,12]. These approaches may prove effective as patients can draw on personal and lived experiences, enabling communication between individuals who share established trust and cultural context [13]. To our knowledge, no studies have specifically engaged patients with HPV-associated oropharyngeal cancer as health educators to promote HPV vaccination. It is plausible that, when equipped with appropriate tools, individuals with HPV-related oropharyngeal cancer could serve as advocates for HPV vaccination within their communities [14,15].

Using a user-centered approach, we previously developed an interactive HPV vaccination education program (IHVEP) with a digital health educator to equip patients with HPV-associated cervical cancer to serve as health educators. In prior pilot trials comparing IHVEP to standard education (written self-help materials) among 76 cervical cancer patients, the program was found to be acceptable, feasible, and potentially effective in promoting vaccine advocacy among patients and uptake among their family and friends [16].

Although HPV contributes a substantial proportion of oropharyngeal cancers, most educational interventions have focused solely on cervical cancer populations. As a result, individuals with HPV-associated oropharyngeal cancer and their social networks may have limited access to tailored educational resources [17]. It remains unclear whether an adapted IHVEP would be valuable to this population.

In this study, we adapted IHVEP to patients with oropharyngeal dysplasia or cancer and conducted user testing to assess content relevance and suitability while identifying factors important to the design of effective digital health interventions. These findings may inform the development of scalable, patient-centered HPV education strategies and contribute to the design of future digital health interventions.

## 2. Materials and Methods

### 2.1. Theoretical Framework

#### 2.1.1. Information-Motivation-Behavioral Skills (IMB) Model

Using a qualitative, user-centered, and theory-based approach, we developed IHVEP (version 1.0), in collaboration with the contracted video company NextThought (Oklahoma city, OK, USA), guided by the Information-Motivation-Behavioral Skills (IMB) model, a framework for promoting health behavior change. The IMB model identifies three key determinants: accurate information, personal and social motivation, and behavioral skills needed to perform a behavior [7]. Within this framework, information and motivation are thought to influence behavior primarily through behavioral skills.

Originally developed to promote HIV preventive behaviors [7], the IMB model has since been applied across multiple disease contexts [18,19] and has demonstrated effectiveness in promoting health behaviors in diverse populations [20,21]. The IMB model has also been shown to support HPV vaccination uptake among young adults [14,22]. Accordingly, the IMB model was used to guide the development of IHVEP content aimed at promoting oropharyngeal cancer prevention behaviors.

#### 2.1.2. Suitability Assessment of Materials (SAM) Tool

The Suitability Assessment of Materials (SAM) is a validated tool for evaluating the quality and appropriateness of patient education materials for specific populations. It has been widely used to guide material development and adaptation across healthcare domains by assessing content, literacy demand, learner engagement, as well as organizational and cultural appropriateness [23,24]. In this study, SAM domains were used to guide the qualitative analysis of user feedback on IHVEP.

### 2.2. Intervention Development

We further developed our cervical cancer–based IHVEP for use among patients with oropharyngeal dysplasia or cancer through an iterative, multi-step process. First, the original program was adapted to include modular content tailored to different cancer types and their association with HPV (e.g., cervical and oropharyngeal cancers), allowing delivery to both patient groups and healthy adults. HPV and vaccination content were revised to reflect oropharyngeal disease and applicability to both sexes, while maintaining cultural relevance across diverse populations. The adapted modules were then produced in collaboration with a video production company. A Spanish version was subsequently developed using translators and voiceover artists to ensure consistency in content and messaging.

**Figure 1:**
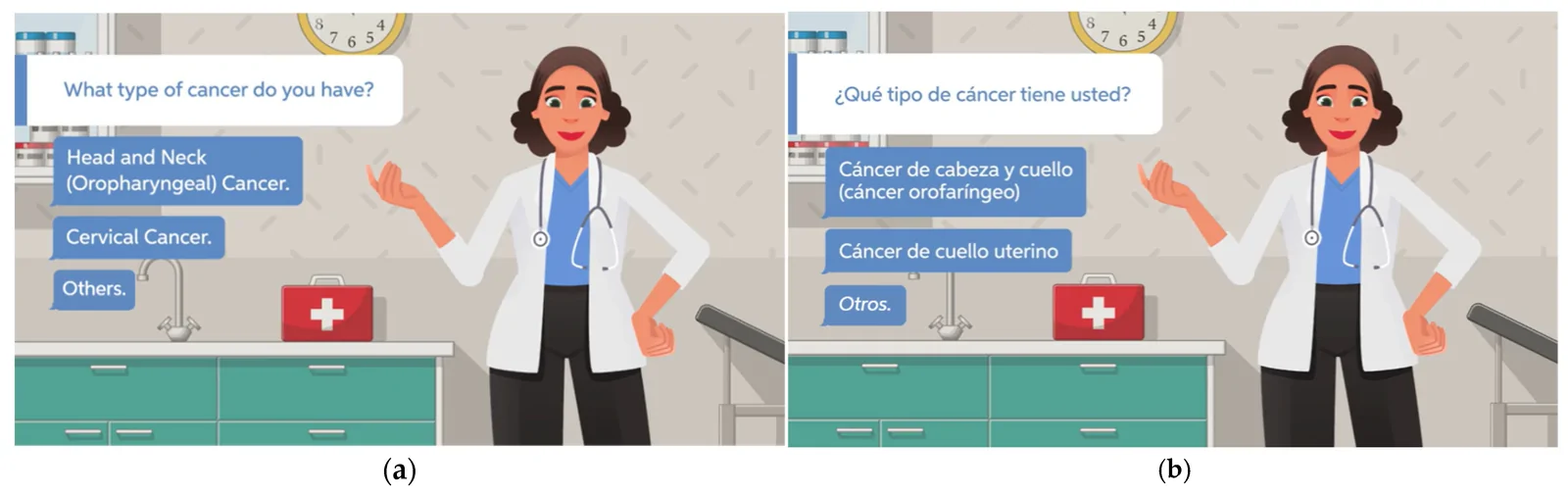
IHVEP interface. (**a**) English version; (**b**) Spanish version.

Most IHVEP content is delivered through an animated physician acting as a digital health educator, with a concluding call to action featuring real physicians encouraging HPV vaccination discussions see Figure 1. The platform uses branching logic to tailor content based on user responses to included questions. Content was expanded to emphasize the link between HPV and cancer and the role of vaccination in prevention, in addition to information and counselling that tap into the IMB-informed communication strategies, including educating the patients about HPV and HPV vaccination, addressing social norms (e.g., getting vaccinated against HPV is the right thing to do to protect themselves and their loved ones), and enhancing perceived behavioral control. IHVEP also has on-demand sections to provide users with information regarding whether the vaccines are safe, what may be some common side effects, where to get more information about the vaccines, where to get the vaccines, and how the vaccination cost may be covered by some insurance or other programs. For patients with oropharyngeal dysplasia or cancer, IHVEP is designed to encourage discussions about HPV vaccination within their social network (primarily their families and friends), supported by communication skill-building elements such as motivational interviewing and active listening. Specifically, IHVEP coaches the patients to start a conversation on this topic by first sharing their fighting-HPV-related cancer story, and then telling their families or friends how to prevent it in a fashion that they have learned from IHVEP (e.g., framing HPV as a cancer-causing rather than a sexually transmitted agent). The patients are also instructed to share the link to IHVEP with their families or friends, so that they can access IHVEP as healthy users and learn more about HPV and HPV vaccination.

### 2.3. Participant Selection

Participants were recruited from head and neck and gynecologic oncology clinics within a single academic health system for a user-testing series to support IHVEP adaptation and provide feedback. During the development of the English version, a multidisciplinary team contributed to content adaptation. For the Spanish version, a professional translator completed the forward translation, followed by a review by two independent Spanish-speaking healthcare providers, who provided feedback and conducted back-translation to identify unclear content.

Once IHVEP reached a near-final version, it was tested among diverse user groups, including healthy individuals, healthcare providers, and patients with oropharyngeal or cervical cancer. Participants reviewed the program and provided feedback based on their experiences. Purposive sampling was used to ensure diversity in age, sex, race/ethnicity, residence, and health literacy. Inclusion criteria were age ≥ 18 years, English or Spanish proficiency, and ability to provide informed consent. Participants were recruited from head and neck and gynecologic oncology clinics within a single academic health system. A total of 12 individuals participated (4 healthy users, 4 healthcare providers, 2 oropharyngeal cancer patients, and 2 cervical cancer patients).

### 2.4. Semi-Structured Interviews

In the final phase, participants completed remote consent and accessed IHVEP via a secure web link on their personal devices. Following program use, one-on-one semi-structured interviews were conducted via Zoom. Open-ended questions, informed by user-centered design principles, were used to elicit feedback on IHVEP and explore perceptions of HPV-related behaviors and prevention. Questions were tailored by participant group: individuals with HPV-associated cancer were asked about their diagnosis and experiences discussing their condition, while healthy participants were asked about community perceptions and their experiences discussing HPV vaccination. Interviews lasted 30–45 min, were conducted in English, and were recorded and transcribed using Zoom (Version: 7.1.0 (41345) for qualitative analysis.

### 2.5. Data Analysis

Qualitative data were analyzed by two reviewers using NVivo software. Feedback was coded and organized into themes aligned with domains of the IMB and SAM frameworks. HPV vaccination-related themes were further analyzed using deductive content analysis, with coding matrices applied to map findings to IMB constructs. Discrepancies were resolved through discussion among the investigative team to achieve consensus. Reporting followed established qualitative research standards to support rigor and transparency.

## 3. Results

### 3.1. Participant Characteristics

Interviewees (12) represented patients with HPV-associated cancers (4), healthy individuals (4), and healthcare providers (4). The majority (75%) of respondents were women. Ages ranged from 21 to 66 years, with an average age of 38 years. Four participants were native Spanish speakers, also bilingual in English, and reviewed both versions of IHVEP. The average time participants spent viewing IHVEP was 16 min.

### 3.2. User Feedback Regarding IHVEP Content

The IMB model’s basic constructs were demonstrated through participants’ opinions of IHVEP content, as described below and summarized in Supplementary Table S1.

#### 3.2.1. Information

The information component of the IMB model was reflected by the participant’s perceived increase in knowledge of HPV prevalence, HPV’s role in cancer pathogenesis, and the HPV vaccine after watching IHVEP. All participants reported that IHVEP was informative and increased their knowledge of HPV.

Through participation with IHVEP, five individuals (who were not healthcare providers) reported newly recognizing the high prevalence of HPV. Across all interviews, expressions of surprise regarding HPV’s widespread occurrence were documented 17 separate times. A 66-year-old male who had battled HPV-associated neck cancer believed that his HPV positive status was a rarity. He was surprised to learn about the prevalence of HPV, stating, “*I don’t think many people realize how prevalent HPV is. Like this video said, most people will come into contact with this virus. And that’s where the cancer down the line starts.*”

All 12 participants reported that IHVEP clearly portrayed the connection between HPV and cancer. Participants remarked how informative IHVEP was 22 different times. A 26-year-old man working in the healthcare field was delighted to be armed with more information regarding HPV and its association with cancer pathogenesis. He remarked, “*Before watching this video, I was probably not quite as knowledgeable as I should have been about the role HPV plays in causing cancer, and I do feel like I was able to learn things in the app, that will be helpful moving forward.*”

A 55-year-old healthy woman discussed how IHVEP provided her with accurate information on the HPV vaccine’s efficacy, safety, and side effects. She believed this information would enable her to more effectively encourage others to get vaccinated. She said, “*Actually, I feel more informed about the HPV vaccine than I did before. I think I could have a decent conversation with others about the benefits of getting the vaccine and the potential side effects.*”

A few responses aligning with the Information construct of the IMBS model were in a negative context when considering the HPV vaccine. For example, misinformation and a lack of the ability to recognize misinformation discouraged one 36-year-old healthy female participant from discussing HPV vaccination with others, even after viewing IHVEP. A 48-year-old woman who had survived HPV-associated cervical cancer was nervous about many side effects of the HPV vaccine that she had seen reported on various social media platforms. She confessed, “*In order for me to recommend others get the HPV vaccine, I would have to see more research about the long-term side effects and information showing me that all of the negative information out there about the side effects is false.*”

#### 3.2.2. Motivation

The motivation construct in the IMB model for recommending HPV vaccination to others was reflected in a desire to protect others, a recommendation from a healthcare provider, and a personal understanding of the consequences of HPV infection. Most users (11/12) reported feeling more motivated to discuss HPV with others after completing IHVEP.

After viewing IHVEP, several participants were concerned about the likelihood of their loved ones contracting HPV and the potential cancers that can result from an HPV infection. One 66-year-old woman who had battled cervical cancer was excited about the vaccine’s ability to prevent cancer in others and exclaimed, “*I’m really concerned about my grandkids. I need to tell them about the HPV vaccine. It’s very important. I wish this were around when I was younger.*”

While most IHVEP content was delivered by an animated physician character, the program concluded with a segment featuring real-life physicians urging viewers to discuss HPV vaccination with their family and friends. Multiple participants remarked that they were highly motivated by actual healthcare providers giving them a call to action. One healthy 22-year-old woman shared, “*I liked that they had three qualified, real-life people talking and not just one animated character. It made it more engaging and motivating.*”

Participants used their personal experience suffering from HPV associated cancers and dysplasia as motivation to discuss HPV vaccination with others. A 55-year-old woman who had undergone treatment for HPV-associated cancer expressed an increased desire to warn others about the dangers of HPV. “*This cancer has been horrible. Knowing we have the HPV vaccine as a tool to prevent it from happening down the line is great. And it’s exciting that we now have the ability to prevent that suffering for others.*”

#### 3.2.3. Behavioral Skills

The behavioral skills pillar of the IMB model was demonstrated by an increase in participants’ objective ability to address and combat stigma surrounding HPV and their self-efficacy for discussing HPV prevention with others after completing IHVEP. While participants valued IHVEP’s coaching, several wished for additional components to further develop their behavioral skills.

All four participants with HPV associated cancer confessed that prior to watching IHVEP, they felt discouraged from discussing HPV with others due to the stigma that surrounds sexually transmitted diseases. Even healthcare providers identified stigma as a limiting factor in discussing HPV in their personal lives. A 27-year-old healthcare provider reported, “*The only reason I would stop discussing HPV is if I’m making the person uncomfortable by talking about sexually transmitted diseases.*”

After watching IHVEP, 83% of participants reported feeling better equipped to navigate sensitive topics surrounding HPV. Users enjoyed the conversational strategies IHVEP provided; some felt that incorporating more enhanced coaching would be impactful. One 55-year-old man who initially felt shame about his HPV positive status was excited to be more empowered to discuss HPV, stating, “*Well, I liked that the program discussed how HPV is so common. The fact that it is so common takes away some of the stigma from talking about it with others.*” He went on to share, “*saying that it is so common helps but it (IHVEP) could be a little more direct about specific ways to get around the stigma.*”

Having the confidence to effectively carry out a conversation about HPV is often a barrier to discussing HPV vaccination. Participants shared that IHVEP equipped them not only with information, but also with conversational strategies they could employ when discussing HPV. A 25-year-old female who did not have experience in discussing preventative healthcare with others shared, “*I now feel very confident in telling my family and friends about HPV. This program gave me both the knowledge and the skills I need to have that conversation.*”

### 3.3. Other User Feedback Regarding Adaptation and Suitability

Guided by the SAM model, we analyzed additional participant feedback to evaluate the adapted IHVEP’s content (applicability and comprehensibility), learner stimulation, literary demand, organization, cultural appropriateness, and overall suitability, as summarized in Supplementary Table S2.

#### 3.3.1. Content Applicability and Comprehensibility

Almost all participants (11/12) felt that the content in IHVEP was both applicable to their lives and provided useful information. One healthy 36-year-old mother who was considering vaccination for her own children remarked, “*You have a little bit of everything in this video. I like that it not only told you why you should consider the preventative but also talked logistically when and where to get the shot. I thought the statistics were useful and the information was very good. I hadn’t gotten all that detailed information compiled in one place. It was more of a big checklist of information that I can use in the future.*”

While users found the information comprehensive and detailed, they did not struggle to understand the material being presented. A healthy 21-year-old noted how the format made it simple to follow along with the complex themes being taught: “*The images were very helpful. I like that there were the little summaries of what they were talking about. So, if I got lost in what they were saying, I could at least follow along visually. Or if someone doesn’t read well, then they could hear it. So it’s kind of like covering all types of learning styles.*”

#### 3.3.2. Learner Stimulation

Users found IHVEP’s interactive format and animations both entertaining and engaging. Participants remarked how stimulating the program was a total of 10 different times. When thinking about how to best educate future generations about the dangers of HPV, a 55-year woman with oropharyngeal cancer shared, “*This program is great, not only for adults, but for kids as well. I think this program would also catch the attention of a younger person too as well. I think that you guys did a pretty good spot on job with making it fun.*”

#### 3.3.3. Literary Demand

Multiple participants (6/8) who were not healthcare providers complained that past conversations with their healthcare providers often involved complex language and medical vocabulary, which hindered their understanding of disease prevention efforts. Regarding IHVEP, all reviewers found that the plain language made it easier to digest. One 22-year-old healthy woman who did not have much prior knowledge of HPV commented, “*I liked that they use, like, simple terms, it wasn’t medical jargon.*”

#### 3.3.4. Organization

Three users specifically commented that the material was organized and presented logically in a clear, straightforward manner. A 55-year-old man who was diagnosed with HPV-associated neck cancer shared that in the past, he was confused about the connection between the virus and his cancer when it was explained to him by his physician, but he had no difficulty following along with the concepts in IHVEP. “*I thought the way the ideas were presented in the program was pretty clear and informative, and yet it was simple to understand.*”

#### 3.3.5. Cultural Appropriateness

Participants were asked to evaluate the cultural appropriateness and relevance of IHVEP. All twelve participants, including native Spanish-speaking participants, reported that IHVEP was appropriate and considerate of their cultural background. They identified no barriers that would inhibit IHVEP from effectively reaching individuals within their specific cultures. Users reported that the images aligned with their culture’s social norms, and many commented that the animated physician felt like a familiar figure. A healthy 25-year-old remarked, “*The character was comforting, soothing, and easy to relate to.*”

#### 3.3.6. Suggested Improvements

We asked users to provide feedback on areas of improvement for IHVEP. Based on the feedback received, necessary adjustments were made to the content, context, and functionality of the digital health intervention. Most suggestions fell into three categories: technology issues, content suggestions, and functionality.

Users reported several minor technical issues with the program interface when attempting to watch IHVEP. One recurring issue was that pop-up blockers had to be disabled to launch IHVEP on a computer. One individual suggested, “*Maybe you can add an asterisk at the beginning that informs users to allow pop-ups or something. That would make the video more accessible.*”

Users recommended additional content they felt could make IHVEP more motivating. A recurring idea was to include a patient with HPV-associated cancer who could share their personal experience with HPV. “*I think it would be a good thing if someone were able to share their story of what HPV has done to their life. It’s always an inspiring thing to hear someone personally talk about their issues with a particular condition.*”

Some participants suggested ways to improve IHVEP functionality to make navigation easier. Many viewers felt they would have learned more information if the program interface were easier to navigate. One participant shared, “*There were a couple times I would have wanted to repeat a slide so that I could hear all the information*; *I would have just had to get a back button.*”

## 4. Discussion

The aim of this study was to evaluate the adaptation of a digital, single-session HPV educational program and assess participant feedback on the revised IHVEP. Originally developed for cervical cancer patients, the program was modified to reflect the experiences of individuals with HPV-associated oropharyngeal cancer. Participants of both genders perceived the adapted IHVEP as informative, motivating, and empowering, with responses aligning with IMB framework constructs. The program appeared to improve understanding of HPV prevalence, its role in cancer pathogenesis, and vaccination. Reported motivators included personal experience with HPV-associated cancer, virtual provider recommendations, and a desire to protect others. The behavioral skills component was associated with increased perceived ability and self-efficacy to promote HPV vaccination, while also identifying areas for further development. These findings suggest that, when provided with appropriate information and support, patients with HPV-associated disease may be able to serve as health educators within their social networks.

The IMB model has been widely used to understand HPV prevention behaviors and has informed several interventions targeting cervical cancer prevention [22,25,26]. These interventions have demonstrated effectiveness in promoting vaccination among young women; however, their applicability to oropharyngeal cancer prevention remains less established. Findings from this pilot study suggest that the IMB framework’s core constructs—information, motivation, and behavioral skills—may also be relevant for HPV vaccination in this context, indicating potential utility for future interventions.

The information component appeared to influence behavior in both facilitating and limiting ways. While most participants reported that IHVEP content encouraged vaccine promotion, some expressed uncertainty regarding its credibility relative to social media sources. This underscores the continued influence of vaccine misinformation and suggests that future interventions may benefit from incorporating training to improve critical evaluation of health information.

Although participants reported increased confidence in discussing HPV, some identified a need for more advanced communication skills training. Suggested enhancements included strategies for initiating conversations, addressing stigma, and navigating sensitive discussions. These findings highlight the importance of communication skills in supporting health behavior promotion and suggest that strengthening behavioral skills components may improve self-efficacy.

SAM-guided qualitative assessment indicated that IHVEP was perceived as relevant, understandable, well-organized, and culturally appropriate. However, participants identified areas for improvement, including navigation, functionality, and motivational content, which may inform future digital health intervention development. Technological usability was also noted as an important factor influencing engagement, with minor issues potentially limiting program completion. Ensuring intuitive design may enhance user experience and effectiveness.

Participants responded positively to the inclusion of physician-delivered content, consistent with prior evidence that provider recommendations are a strong motivator for HPV vaccination [8]. Findings from this study suggest that virtual provider recommendations may also influence attitudes toward HPV vaccination. Participants additionally expressed interest in incorporating personal testimonials from individuals affected by HPV-associated cancers. While animated components were well received, inclusion of real individuals may enhance credibility and emotional engagement, potentially strengthening motivation.

Raising awareness of HPV-related risks remains a public health challenge, as recent data indicate awareness of the link between HPV and certain cancers is limited among adults [27]. By increasing awareness and providing practical vaccination information, IHVEP may help equip patients to share knowledge within their communities, potentially contributing to increased vaccination uptake and reduced disease burden. While prior interventions have engaged patients with chronic diseases as peer educators, few have explored this approach among individuals affected by HPV-associated cancers [10,11]. Findings from this pilot study suggest participants perceived IHVEP as informative and expressed motivation to share it with others, indicating potential for such interventions to support patients in their roles as community health advocates.

A key strength of this study is the inclusion of both English- and Spanish-speaking participants, enhancing cultural relevance. However, several limitations should be noted, including the small sample size and recruitment from a single urban center, which may limit generalizability. Although participants represented diverse cultural backgrounds, geographic diversity was limited. Future studies with larger and more diverse populations are needed to further evaluate the feasibility and effectiveness of the adapted IHVEP.

## 5. Conclusions

This pilot study evaluated a theory-based, single-session HPV educational video adapted for oropharyngeal cancer patients. Participants, including healthcare providers, healthy individuals, and patients with HPV-associated cancers, provided feedback through semi-structured interviews. Qualitative analysis guided by the IMB framework suggests that IHVEP may enhance perceived knowledge of HPV, increase motivation to promote vaccination, and improve self-efficacy in discussing HPV prevention. Participants generally found the program to be clear, engaging, and appropriate for the target population. Areas for improvement included enhancing skills for evaluating health information, improving usability, and incorporating content featuring real individuals.

Lessons learned from this development provide preliminary support for the continued refinement of IHVEP and may inform future digital interventions to encourage disease-prevention behaviors. With further validation, such interventions may help equip patients with the knowledge, motivation, and skills needed to engage in health promotion within their social networks, potentially contributing to increased vaccination uptake and reduced disease burden.

## Data Availability

Data are available from the corresponding author upon reasonable request and in accordance with the University of Oklahoma Health Sciences Center (OUHC) data-sharing policies and applicable data use agreements.

## Supplementary Materials

The following supporting information can be downloaded at: https://www.mdpi.com/article/10.3390/vaccines14080689/s1, Table S1: Participant’s Response Supporting Information, Motivation, and Behavioral Skills Themes; Table S2: Participants’ Response Regarding the Adaptation and Suitability of IHVEP.

## Abbreviations

The following abbreviations are used in this manuscript:

HPV	Human Papillomavirus
IHVEP	Interactive Human Papillomavirus Education Program
IMB	Information-Motivation-Behavioral Skills Model
SAM	Suitability Assessment of Materials
US	United States
CDC	Centers for Disease Control and Prevention
COVID-19	Coronavirus Disease 2019
HIV	Human Immunodeficiency Virus
