# Supplementary material for: Developing an Interactive Human Papillomavirus Vaccination Education Program for Oropharyngeal Cancer Patients and Healthcare Providers: Lessons Learned from the Target Users’ Feedback
Source: Vaccines (Basel). 2026 Aug 11;14(8):689. doi: 10.3390/vaccines14080689 (PMC13517443; doi:10.3390/vaccines14080689)
Supplement: Supplementary file 1 [file vaccines-14-00689-s001.zip › vaccines-4399020-supplementary.pdf]

Table S1

Participant's Response Supporting Information, Motivation, and Behavioral Skills Themes

Themes	Participants	Participant's Response (Quote)
Information	66-years-old male, HPV-associated neck cancer survivor	*I don't think many people realize how prevalent HPV is. Like this video said, most people will come into contact with this virus. And that's where the cancer down the line starts.* "
Information	26-year-old male, healthcare provider	"*Before watching this video, I was probably not quite as knowledgeable as I should have been about the role HPV plays in causing cancer, and I do feel like I was able to learn things in the app, that will be helpful moving forward"*
Information	55-year-old healthy woman	"*Actually, I feel more informed about the HPV vaccine than I did before. I think I could have a decent conversation with others about the benefits of getting the vaccine and the potential side effects."*
Information	48-year-old cervical cancer survivor	*"In order for me to recommend others get the HPV vaccine, I would have to see more research about the long-term side effects and information showing me that all of the negative information out there about the side effects is false."*
Motivation	66-year-old cervical cancer survivor	*"I'm really concerned about my grandkids. I need to tell them about the HPV vaccine. It's very important. I wish this were around when I was younger."*
Motivation	22-year-old healthy woman	*"I liked that they had three qualified, real-life people talking and not just one animated character. It made it more engaging and motivating."*
Motivation	55-year-old woman, HPV-associated cancer survivor	"*This cancer has been horrible. Knowing we have the HPV vaccine as a tool to prevent it from happening down the line is great. And it's exciting that we now have the ability to prevent that suffering for others."*
Behavioral Skills	27-year-old healthcare provider	"*The only reason I would stop discussing HPV is if I'm making the person uncomfortable by talking about sexually transmitted diseases."*
Behavioral Skills	55-year-old male	"*Well, I liked that the program discussed how HPV is so common. The fact that it is so common takes away some of the stigma from talking about it with others."* He went on to share, "*saying that it is so common helps but it (IHVEP) could be a little more direct about specific ways to get around the stigma"*
Behavioral Skills	25-year-old healthy woman	*"I now feel very confident in telling my family and friends about HPV. This program gave me both the knowledge and the skills I need to have that conversation."*

Table S2

Participants' Response Regarding the Adaptation and Suitability of IHVEP

**Themes**	**Participants**	**Participant's Response (Quote)**
Content Applicability and Comprehensibility	36-year-old healthy mother	*"You have a little bit of everything in this video. I like that it not only told you why you should consider the preventative but also talked logistically when and where to get the shot. I thought the statistics were useful and the information was very good. I hadn't gotten all that detailed information compiled in one place. It was more of a big checklist of information that I can use in the future."*
Content Applicability and Comprehensibility	21-year-old healthy participant	*"The images were very helpful. I like that there were the little summaries of what they were talking about. So, if I got lost in what they were saying, I could at least follow along visually. Or if someone doesn't read well, then they could hear it. So it's kind of like covering all types of learning styles."*
Learner Stimulation	55-year women with oropharyngeal cancer	*"This program is great, not only for adults, but for kids as well. I think this program would also catch the attention of a younger person too as well. I think that you guys did a pretty good spot on job with making it fun."*
Literary Demand	22-year-old healthy woman	"*I liked that they use, like, simple terms, it wasn't medical jargon.*"
Organization	55-year-old man, diagnosed with HPV-associated oropharyngeal cancer	"*I thought the way the ideas were presented in the program was pretty clear and informative, and yet it was simple to understand."*
Cultural Appropriateness	Healthy 25-year-old participant	*"The character was comforting, soothing, and easy to relate to."*
Suggested Improvements	Multiple Participants	*"Maybe you can add an asterisk at the beginning that informs users to allow pop-ups or something. That would make the video more accessible."*<br>*"I think it would be a good thing if someone were able to share their story of what HPV has done to their life. It's always an inspiring thing to hear someone personally talk about their issues with a particular condition."*<br>"*There were a couple times I would have wanted to repeat a slide so that I could hear all the information; I would have just had to get a back button."*
